# Annexin A2 is not a good biomarker for hepatocellular carcinoma in cirrhosis

**DOI:** 10.3892/ol.2013.1337

**Published:** 2013-05-08

**Authors:** ZHIKUN LIU, QI LING, JIANGUO WANG, HAIYANG XIE, XIAO XU, SHUSEN ZHENG

**Affiliations:** 1 Key Laboratory of Combined Multi-Organ Transplantation, Ministry of Public Health, Zhejiang University School of Medicine, Hangzhou, Zhejiang 310003, P.R. China; 2 Department of Surgery, Division of Hepatobiliary and Pancreatic Surgery, First Affiliated Hospital, Zhejiang University School of Medicine, Hangzhou, Zhejiang 310003, P.R. China

**Keywords:** annexin A2, hepatocellular carcinoma, liver cirrhosis, liver transplantation, prognosis

## Abstract

In China, hepatocellular carcinoma (HCC) usually develops following a long history of chronic hepatitis B infection or cirrhosis. To evaluate the diagnostic role of annexin A2 (ANXA2), a possible tumor marker, in patients with hepatitis B virus (HBV)-related HCC, particularly those with a history of cirrhosis, the present study prospectively enrolled 87 patients with HBV-related HCC (with cirrhosis), 39 patients with HBV-related cirrhosis and 27 healthy controls. The expression levels of serum and tissue ANXA2 were determined using an enzyme-linked immunosorbent assay (ELISA) and immunohistochemical staining, respectively. The serum levels of ANXA2 were significantly elevated in the patients with HCC (median, 567.2 *μ*g/ml; P=0.003) and cirrhosis (median, 414.8 *μ*g/ml; P=0.011) compared with the healthy controls (median, 241.9 *μ*g/ml). However, no significant differences were observed in the serum ANXA2 levels between the patients with HCC and those with cirrhosis. The immunohistochemical staining analysis showed that the healthy controls did not show positive staining, while the number of cases immunoreactive for ANXA2 steadily increased from the liver cirrhosis tissues (20/39, 51.3%) to the non-cancer (53/87, 60.9%) and cancer tissues (68/87, 78.2%). The cancer tissues exhibited a significantly higher ANXA2-positive rate compared with the non-cancer (P=0.013) and liver cirrhosis tissues (P=0.002). Furthermore, marked ANXA2 staining was more prevalent in the cancer tissues (16/87, 18.4%) than the non-cancer (4/87, 4.6%; P=0.004) and liver cirrhosis (1/39, 2.6%; P=0.034) tissues. The sensitivity, specificity and diagnostic accuracy of tissue ANXA2 for HCC in cirrhosis were 78.2, 42.1 and 56.8%, respectively. The ANXA2 expression levels in the serum and cancer tissues were not associated with tumor-free survival or patient survival following liver transplantation. Serum or tissue ANXA2 is not a good diagnostic marker for HCC in HBV-related cirrhosis and is not associated with prognosis.

## Introduction

Hepatocellular carcinoma (HCC) is a major form of liver cancer and has become the fifth most common malignancy and the third leading cause of cancer-related mortality worldwide (1,2). Hepatitis B virus (HBV)- or hepatitis C virus (HCV)-induced liver cirrhosis is a significant risk factor for HCC as >80% of HCCs feature a history of cirrhosis (3). Due to the particularly high prevalence of HBV infection in the Chinese population, HBV-related liver disease (e.g. chronic hepatitis, cirrhosis and HCC) is one of the major healthcare burdens in China. There are an estimated 30 million individuals chronically infected with HBV. During a 5-year period, 10% of these patients developed cirrhosis and 6.5% of the cirrhotic patients suffered from HCC (4). Although surgical treatment (hepatic resection and transplantation) is the most effective therapy, it is associated with high tumor recurrence rates and consequently poor prognoses (5). Furthermore, numerous cases are not suitable for surgery as the diseases are at the end-stage. Therefore, it is important to identify biomarkers for HCC to enable the early diagnosis and monitoring of tumor recurrence.

Annexin A2 (ANXA2), a member of the annexin family, is involved in various biological functions including cell-cell adhesion (6), cell proliferation (7,8), cell surface fibrinolysis (9,10), cell growth regulation and apoptosis (11–13) and the cocarcinogenic effects of progastrin (14). It has been reported that ANXA2 is frequently upregulated in HCC patients compared with controls (15,16), suggesting that ANXA2 is associated with HCC. In addition, ANXA2 has been demonstrated to be a potential biomarker of immune liver fibrosis in a rat liver fibrosis model (16), indicating that ANXA2 may also be associated with liver fibrosis or even cirrhosis.

The primary objective of the present study was to clarify whether ANXA2 is a useful biomarker for distinguishing HCC from cirrhosis; the secondary objective was to evaluate the role of ANXA2 in predicting HCC recurrence following liver transplantation.

## Materials and methods

### Patients and samples

A total of 130 adult patients with HBV-related HCC (with cirrhosis; n=88) and HBV-related cirrhosis (n=42) were prospectively enrolled between October 2006 and January 2008 at the First Affiliated Hospital, Zhejiang University School of Medicine, Hangzhou, Zhejiang, China. All patients received a primary liver transplantation and anti-HBV treatment according to the standard protocol (17). Patients with HCC fell within the Hangzhou criteria (18). A total of four patients were excluded as three cases suffered early mortality due to hemorrhaging following transplantation and one was lost to follow-up. The healthy controls (n=27) were donors for living donor liver transplantation. The patient characteristics are shown in Table I. The median follow-up time was 52 months (range, 14–74 months).

Peripheral venous blood samples were obtained prior to surgery. Serum samples were allowed to clot and stored at −80°C. The tissues were paraffin-embedded following fixation in 10% formalin for 24–48 h. The non-cancer tissues were the cirrhotic tissues surrounding the cancer tissue. All surgeries were performed by the same group led by Professor Shusen Zheng. HCC and cirrhosis were diagnosed by imaging examinations and biopsy and verified by histological examination following surgery.

Informed consent was obtained from each patient. The present study was approved by the Ethics Committee of the First Affiliated Hospital, Zhejiang University, and performed strictly according its guidelines, the regulations of the Organ Transplant Committee of Zhejiang province and the Declaration of Helsinki.

### Enzyme immunoassay

Serum ANXA2 levels were determined using a sensitive enzyme immunoassay (ANXA2 kit; Uscn Life Science Inc., Wuhan, China) according to the manufacturer’s instructions. Briefly, 100 *μ*l of standard, blank or samples were added into the appropriate wells, which were precoated with a monoclonal antibody specific for ANXA2, and incubated at 37°C for 2 h. Detection reagent A working solution was added to each well for 1 h at 37°C, then 100 *μ*l of detection reagent B working solution was added to each well for 30 min at 37°C. At 10 min, following color development, the intensity was read at 450 nm. The results were calculated from a standard curve (recombinant human ANXA2; range, 0.625–40 ng/ml) generated from a four-parameter logistic curve fit. Measurements were performed in duplicate and the mean values were taken.

### Immunohistochemical staining

Immunohistochemical staining was performed as described previously (19). The cases were semi-quantitatively evaluated with a four-tiered system by two independent pathologists and assessed using the immunoreactive score (IRS) (20). Individual cases were considered immunoreactive (IR) for antigens when >10% of the cells were stained. The percentage of positive cells was assessed semi-quantitatively: 0, absent; 1, <10% of positive cells; 2, 10–49%; 3, 50–80%; and 4>80%. To achieve the final score, the quantity of immunoreactive cells was multiplied by the staining intensity: 0, absent; 1, weak; 2, moderate; and 3, marked. The final score was considered as follows: 0, negative; 1–4, +; 5–8, ++; and 9–12, +++. The staining assessment was performed by two independent pathologists. The concordance on agreed scores was achieved with a high k coefficient value (>0.80).

### Statistical analysis

Categorical data are presented as a number and percentage, while continuous data are presented as the mean and standard deviation or median (25th to 75th percentile of the inter-quartile range). Categorical data were compared using the Chi-squared test and continuous data were compared with the Student’s t-test or Mann-Whitney test. A Spearman’s rank correlation was performed to analyze the correlation. The cutoff value was selected according to receiver-operating characteristic curves. P<0.05 was considered to indicate a statistically significant difference. Interobserver variability was assessed with the κ index. All statistical analyses were performed using SPSS 13.0 software (SPSS, Chicago, IL, USA).

## Results

### Serum ANXA2 for the diagnosis of HCC

**Figure 1.**
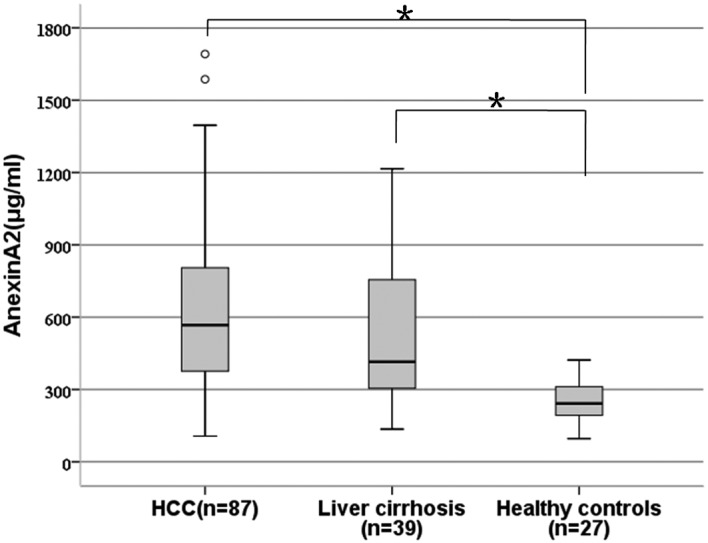
Serum annexin A2 (ANXA2) expression in HCC, cirrhosis and healthy control tissues. ^*^Statistically significant difference revealed by multiple pairwise comparisons between healthy controls and liver cirrhosis (P= 0.011) and healthy controls and HCC (P= 0.003). °Outliers. HCC, hepatocellular carcinoma.

The serum levels of ANXA2 were significantly increased in the patients with HCC (median, 567.2 vs. 241.9 *μ*g/ml; P=0.003) and cirrhosis (median, 414.8 *μ*g/ml vs. 241.9 *μ*g/ml, P=0.011) compared with the healthy controls (Fig. 1). However, there was no significant difference in the serum ANXA2 levels between the patients with HCC and those with cirrhosis (P=0.342).

There was no statistical association between serum ANXA2 and age, gender, tumor differentiation or tumor size (data not shown).

### Tissue ANXA2 for the diagnosis of HCC

**Figure 2.**
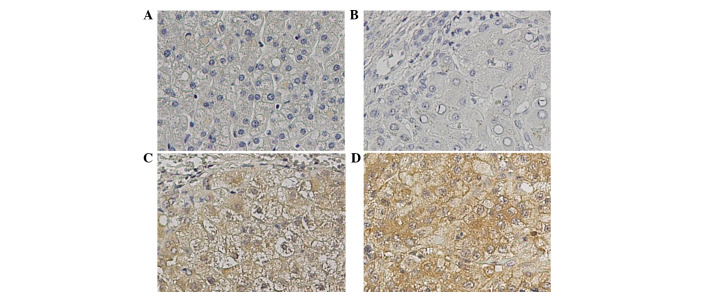
Tissue Annexin A2 expression (immunohistochemistry; magnification, ×100). (A) Healthy control tissue; (B) liver cirrhosis; (C) non-cancer tissue showing diffuse and moderate cytoplasmic and membranous staining; (D) cancer tissue showing diffuse and marked cytoplasmic and membranous staining.

**Figure 3.**
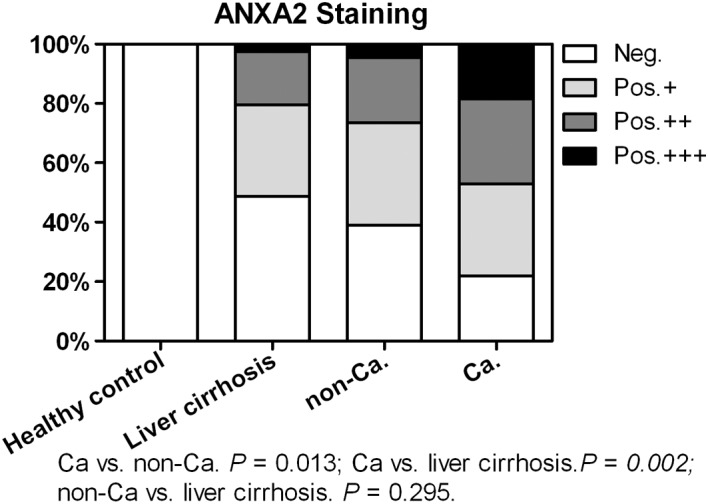
ANXA2 immunohistological testing in healthy control, liver cirrhosis, non-cancer (non-Ca.) and cancer (Ca.) tissues. ANXA2, annexin A2.

ANXA2 protein in the tissue was expressed either at the cell membrane or in the cytoplasm of cirrhotic and tumor cells (Fig. 2). Healthy controls were not immunostained while the number of cases immunoreactive for ANXA2 steadily increased from the liver cirrhosis tissues (20/39, 51.3%) to the non-cancer (53/87, 60.9%) and cancer (68/87, 78.2%; Fig. 3) tissues. The cancer tissues exhibited a significantly higher ANXA2-positive rate than the non-cancer (P=0.013) and liver cirrhosis tissues (P=0.002). Furthermore, the cancer tissues (16/87, 18.4%) showed a higher marked ANXA2 staining rate (+++) compared with the non-cancer (4/87, 4.6%, P=0.004) and cirrhosis (1/39, 2.6%, P=0.016) tissues. There was no significant difference between the non-cancer and liver cirrhosis tissues (P=0.295).

Considering the healthy controls and the cirrhosis and non-cancer tissues as non-malignant, the sensitivity, specificity and accuracy of ANXA2 for HCC detection were 78.2, 52.3 and 61.7%, respectively. Considering only the cirrhosis and non-cancer tissues as non-malignant, the sensitivity, specificity and diagnostic accuracy of ANXA2 were 78.2, 42.1 and 56.8%, respectively.

There was no statistical association between the positive expression of tissue ANXA2 and age, gender, tumor differentiation, tumor size or HCC recurrence.

### Association between serum and tissue ANXA2 levels

A significant correlation was identified between the serum and cancer tissue ANXA2 levels (r=0.364, P=0.017), but not between the serum and the non-cancer tissues (r=0.243, P=0.160) in the HCC patients. A significant correlation was also observed between the serum and tissue ANXA2 levels in the cirrhotic patients (r=0.312, P=0.023).

### Tissue/serum ANXA2 and prognosis in HCC patients

The serum ANXA2 levels did not differ significantly between the patients with HCC recurrence and those without HCC recurrence following liver transplantation (P>0.05). Patient survival did not differ significantly between the patients with high serum ANXA2 expression and those with low expression (P>0.05). Additionally, no significant differences were observed in patient survival or tumor-free survival between the positive expression of tissue ANXA2 and the negative expression of tissue ANXA2 (P>0.05).

## Discussion

In the present study, a typical multistage hepatocarcinogenesis model (healthy, cirrhosis and HCC) was used to evaluate the roles of serum and tissue ANXA2 in the diagnosis of HCC. In accordance with the previous study (15), the results demonstrated that the analysis of serum ANXA2 was able to discriminate between the HCC and healthy patients. However, the serum ANXA2 levels were also clearly elevated in the patients with cirrhosis compared with the healthy controls. Furthermore, no significant differences were observed in the serum ANXA2 levels between the patients with HCC and those with cirrhosis, indicating that ANXA2 was not a good serological diagnostic marker for HCC, particularly in patients with a history of cirrhosis. ANXA2 may serve as a biomarker for liver cirrhosis.

Ultrasound-guided fine-needle aspiration biopsy is a much safer and less traumatic procedure than open surgery in the diagnosis of cancer. The acquired histopathological results may guide the therapeutic strategy and predict a patient prognosis (21). In the present study, the diagnostic and prognostic role of tissue ANXA2 in HCC was assessed. The results revealed that the positive expression rate of ANXA2 was significantly higher in the cancer tissues compared with the cirrhotic and normal liver tissues, suggesting that the analysis of tissue ANXA2 was more likely to distinguish HCC from non-malignant cirrhotic tissues than the analysis of serum ANXA2. Although tissue ANXA2 had a high sensitivity, the specificity was extremely low, contributing to a low diagnostic accuracy. Therefore, neither serum nor tissue ANXA2 would be a good biomarker for HCC patients with a history of cirrhosis. Furthermore, it was observed that the tissue ANXA2 levels were not associated with tumor-recurrence and mortality following liver transplantation, indicating that the early detection of ANXA2 in cancer tissues by biopsy does not aid in the prediction of a patient prognosis.

In addition, there were no significant associations between the expression levels of serum or tissue ANXA2 and the tumor characteristics, including size, number and differentiation. A marked correlation was observed between the serum and tissue ANXA2 levels in the patients with HCC and cirrhosis. The present results provided further evidence that ANXA2 may not be a unique product of tumors but that it is also produced by cirrhotic tissue. Several previous studies have demonstrated that ANXA2 is differentially expressed between normal tissues and tissues of liver fibrosis induced by various causes (e.g. alcohol, the immune system or HBV) (16,22–24). Certain researchers have even considered ANXA2 to be an early effector molecule during the progression of fibrosis (22). Therefore, ANXA2 may not be produced and released by cancer only and it is therefore not a valid biomarker.

In conclusion, the expression of serum and tissue ANXA2 was not only elevated in the patients with HBV-related HCC but also in the patients with liver cirrhosis. ANXA2 expression is not a good serological or immunological diagnostic marker for differentiating HCC from cirrhosis. In the HCC patients, ANXA2 in the serum or cancer tissues was not associated with prognosis following liver transplantation.

## Figures and Tables

**Table I.** Patient characteristics.

Characteristic	HCC (n=87)	Cirrhosis (n=39)	Healthy controls (n=27)
Age (years)	48.1±8.6	45.5±5.1	31.5±4.7
Male/female, n	66/21	29/10	18/9
HBV DNA (+), n	48	21	-
HBV relapse, n	18	9	-
MELD score	12.1±7.4	19.1±5.2	-
Tumor size (cm), n			
≤5	51	-	-
5< n ≤8	22	-	-
>8	14	-	-
Multiple tumors, n	40	-	-
Tumor differentiation, n			
Good	11	-	-
Moderate	76	-	-
Poor	0	-	-
AFP (≥20 ng/ml), n	66	-	-

Age and MELD scores are presented as mean ± SD. HCC, hepatocellular carcinoma; HBV, hepatitis B virus; MELD, Model for End-Stage Liver Disease; AFP, α-fetoprotein.
